# A valuable computed tomography-based new diagnostic tool for severe chest lesions in active pulmonary tuberculosis: combined application of influencing factors

**DOI:** 10.1038/s41598-020-59041-z

**Published:** 2020-02-06

**Authors:** Kui Li, Zicheng Jiang, Yanan Zhu, Chuanqi Fan, Tao Li, Wenqi Ma, Yingli He

**Affiliations:** 1 Department of Infectious Diseases, The First Affiliated Hospital of Xi’an Jiaotong University, 277 West Yanta Road, Xi’an, 710061 Shaanxi China; 2 Department of Infectious Diseases, Ankang Central Hospital, 85 South Jinzhou Road, Ankang, 725000 Shaanxi China; 3 The Medical Imaging Centre, Ankang Central Hospital, 85 South Jinzhou Road, Ankang, 725000 Shaanxi China; 4 Department of Ultrasound, The Second Affiliated Hospital of Xi’an Jiaotong University, 157 West 5 Road, Xi’an, 710004 Shaanxi China

**Keywords:** Experimental models of disease, Risk factors

## Abstract

The objective of the present investigation was to explore the influencing factors and value of computed tomography (CT) for diagnosing severe chest lesions in active pulmonary tuberculosis (APTB). This retrospective investigation included 463 patients diagnosed with APTB. Relevant clinical features were collected. Patients were assigned to mild/moderate group or advanced group depending on the lesion severity on chest CT, severe chest CT lesion refers to the moderately dense or less diffuse lesion that exceeds the total volume of one lung, or the dense fusion lesion greater than one third of the volume of one lung, or the lesion with cavity diameter ≥4 cm. Independent risk factors for severe lesions were determined by univariate and multivariate logistic regression analyses, and the diagnostic efficiency of the risk factors was assessed by receiver operating characteristic curve (ROC). Chest CT demonstrated that there were 285 (61.56%) cases with severe lesions; multivariate Logistic regression analysis showed dust exposure [odds ratio (OR) = 4.108, 95% confidence interval (CI) 2.416–6.986], patient classification (OR = 1.792, 95% CI 1.067–3.012), age (OR = 1.018, 95% CI 1.005–1.030), and albumin-globulin ratio (OR = 0.179, 95% CI 0.084–0.383) to be independently correlated with severe lesions on chest CT. ROC curve analysis showed the cutoff values of age, albumin-globulin ratio and combined score to be 39 years, 0.918 and −0.085, respectively. The predictive value of combined score area under the curve 0.753 (95% CI 0.708–0.798) was higher than that of any single factor. The combined score of these four factors further improved the predictive efficacy.

## Introduction

Tuberculosis is still the most lethal infectious disease in the world. Computed tomography (CT) examination is more and more broadly applied in the diagnosis of the patients with tuberculosis, which is not only the main tool to evaluate the efficacy and severity of disease treatment^1^, but also the main tool to analyze the relationship between extensive lesion in lungs and mortality^2^. However, it is not accessible in grassroots prevention and control institutions of tuberculosis because the equipment is expensive. Thus, it is valuable for clinicians to explore the predictors of severe lesions in chest CT to understand the degree of intrapulmonary lesions more accurately, especially in the absence of CT examination results.

Previous investigations reported that imaging progressive lesions were correlated with the increase of platelet count and thrombocytocrit^3,4^, cellular immune function^5,6^, low albumin^7^ and smoking^8–10^, but inconsistent with correlation reports of diabetes mellitus^7,11–13^ and sputum bacterial load^7,14,15^. These investigations mainly employed univariate analysis, in which not only the diagnostic efficiency was low, but also the results were inconsistent. Therefore, further research is urgently needed. In the present investigation, the patients with active pulmonary tuberculosis (APTB) diagnosed by chest CT were selected as the research subjects to explore the risk factors and diagnostic value of severe lesions in chest CT examinations in order to provide convenient and rapid evaluation index for clinic.

## Results

### General characteristics of APTB patients

There were 285 cases with severe lesions of the chest CT examination in 463 patients with hospitalization, accounting for 61.56%; and there were 178 cases with mild to moderate lesions, accounting for 38.44%. The characteristics of the severe lesions of the chest CT results were illustrated in Table 1.

**Table 1:** Main characteristics of the severe lesions examined by chest CT examination.

CT findings	Number (*n* = 285), n (%)
Patch-like shadow	14 (4.91)
Filamentous shadow	33 (11.58)
Cavity	206 (72.28)
Thick-walled cavity	116 (56.31)
Mouth-eaten cavity	51 (24.76)
Thin-walled cavity	9 (4.37)
Mixed cavity	30 (14.56)
Speckled nodular shadow	17 (5.96)
Cheese-like lesion	12 (4.21)
Other lesions^a^	3 (1.06)

CT: Computed tomography. ^a^Refer to the patients with almost no lesion in the lung, mainly pleural effusion, and mycobacterium tuberculosis deoxyribonucleic acid or ribonucleic acid positive pleural effusion.

### The difference of the influencing factors on the degree of lesion in chest CT

**Figure 1:**
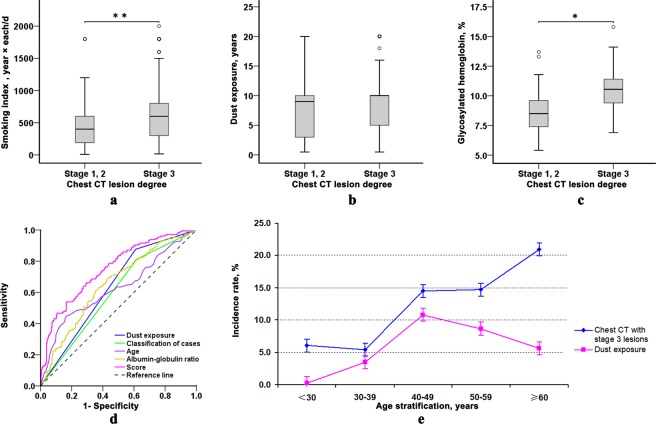
The comparison results of chest CT lesion degree in the patients with active pulmonary tuberculosis between smoking index (**a**), dust exposure time (**b**) and glycosylated hemoglobin (**c**). Score and single risk factor were used to evaluate the working characteristic curve of CT results of severe lesions (**d**). Age and score were supplemented by 100 and 10, respectively. Trend of incidence of severe lesions of chest CT and dust exposure rate with age (**e**). *p < 0.05; **p < 0.01.

Univariate analysis data demonstrated that there were no significant differences in sex, extrapulmonary tuberculosis, diabetes, drug-resistant tuberculosis, sputum bacterial load before treatment (grade), body mass index, platelet, CD4 and CD8 T lymphocytes between the two groups (P > 0.05), while there were significant differences in age, smoking history, dust exposure history, patient classification, duration of symptoms, leukocyte, hematocrit, erythrocyte sedimentation rate, albumin, globulin and albumin-globulin ratio (P < 0.05) (Tables 2, 3). There were significant differences in the smoking index in the smoking population (P = 0.001) (Fig. 1a). In the people exposed to dust, there was no significant difference in the cumulative exposure years between the two groups (P = 0.295) (Fig. 1b). The level of the glycosylated hemoglobin in the diabetic population was statistically significant between the two groups (P = 0.010) (Fig. 1c). The data were illustrated in Table S1.

**Table 2:** Univariate analysis of chest CT lesion degree (nominal and ordinal variables).

Variable		Stage 1, 2 APTB (n = 178)	Stage 3 APTB (n = 285)	*χ*^2^ value	*P* value
Sex, n (%)	Female	45 (25.28)	55 (19.30)	2.316	0.128
	Male	133 (74.72)	230 (80.70)		
Smoking history, n (%)	No	90 (50.56)	95 (33.33)	13.556	<0.001^*^
	Yes	88 (49.44)	190 (66.67)		
Dust exposure history, n (%)	No	156 (87.64)	174 (61.05)	37.831	<0.001^*^
	Yes	22 (12.36)	111 (38.95)		
Classification of cases, n (%)	Initial treatment^a^	145 (81.46)	177 (62.11)	19.381	<0.001^*^
	Retreatment^b^	33 (18.54)	108 (37.89)		
Extrapulmonary tuberculosis, n (%)	No	154 (86.52)	235 (82.46)	1.345	0.246
	Yes	24 (13.48)	50 (17.54)		
Diabetes mellitus, n (%)	No	157 (88.20)	263 (92.28)	2.163	0.141
	Yes	21 (11.80)	22 (7.72)		
Drug-resistant tuberculosis, n (%)	No	163 (91.57)	249 (87.37)	1.976	0.160
	Yes	15 (8.43)	36 (12.63)		
Grade^c^, n (%)	DNA/RNA positive^d^	42 (23.60)	62 (21.75)	3.065	0.690
	NC	13 (7.30)	14 (4.91)		
	1+	50 (28.09)	75 (26.32)		
	2+	24 (13.48)	47 (16.49)		
	3+	34 (19.10)	54 (18.95)		
	4+	15 (8.43)	33 (11.58)		

APTB: active pulmonary tuberculosis; CT: computed tomography; NC: The number of colony. ^a^New cases are defined as not starting anti-TB treatment or being on anti-TB treatment for <1 month; ^b^Previously treated cases are defined as those anti-TB treated ≥1 month in the past; ^c^Smear grading before treatment; ^d^Mycobacterium tuberculosis deoxyribonucleic acid or ribonucleic acid positive; ^*^Significant influence.

**Table 3:** Univariate analysis of chest CT lesion degree (numeric variables).

Variable	Stage 1, 2 APTB (n = 178)		Stage 3 APTB (n = 285)		Test value	*P* value
	Missing data n (%)	Median (interquartile range)^a^	Missing data n (%)	Median (interquartile range)^a^		
Age (years)	0	45.00 (26.75–60.00)	0	53.00 (42.00–64.50)	31795.50	<0.001^*^
Body mass index (kg/m^2^)	3 (1.69)	19.267 (17.928–21.223)	2 (0.70)	19.111 (17.313–20.957)	22619.00	0.119
Duration of symptoms (days)	0	60.00 (15.00–183.00)	0	183.00 (30.00–1095.00)	33495.50	<0.001^*^
White blood cell (×10^9^/L)	0	6.29 (5.15–7.95)	0	7.19 (5.57–8.98)	29818.00	0.001^*^
Hematocrit (%)	0	38.00 ± 6.21	0	36.42 ± 6.16	2.682	0.008^*^
Platelet (×10^9^/L)	0	247.00 (194.00–315.25)	0	279.00 (208.50–346.50)	28635.00	0.020^*^
ESR (mm/H)	15 (8.43)	40.00 (21.00–68.00)	32 (11.23)	58.00 (33.00–78.00)	25879.50	<0.001^*^
Albumin (g/L)	0	32.49 ± 5.61	0	29.58 ± 6.07	5.176	<0.001^*^
Globulin (g/L)	3 (1.69)	31.34 (27.81–35.07)	2 (0.70)	32.93 (29.50–36.96)	29100.50	0.002^*^
Albumin-globulin ratio	3 (1.69)	1.036 (0.859–1.288)	2 (0.70)	0.879 (0.719–1.083)	17218.50	<0.001^*^
CD4 T lymphocytes (cells/μL)	32 (17.98)	317.50 (250.00–401.50)	40 (14.04)	293.00 (196.50–419.50)	16090.00	0.097
CD8 T lymphocytes (cells/μL)	32 (17.98)	274.50 (189.75–382.75)	40 (14.04)	258.00 (170.50–349.50)	16524.00	0.208

APTB: active pulmonary tuberculosis; CT: computed tomography; ESR: erythrocyte sedimentation rate. ^a^Data are presented as the value of variable; ^*^Significant influence.

### Combined risk factors predicted severe lesions of chest CT

Seventeen variables were included in the Logistic regression analysis with the adjusted test level as the criterion. The results exhibited that dust exposure, patient classification, age, albumin-globulin ratio were independent risk factors, and duration of symptoms was not statistically significant, as demonstrated in Table 4. The Logistic regression model served as a new variable, called “SCORE”, which was defined as follows:

**Table 4:** Logistic regression analysis of chest CT lesion degree.

Variable	*β*-coefficient	${S}_{\bar{x}}{\rm{value}}$	*Wald χ*^2^ value	*P* value	Odds ratio	95% *CI*
Intercept	0.706	0.513	1.894	0.169	2.026	
Dust exposure	1.413	0.271	27.218	<0.001^*^	4.108	2.416–6.986
Classification of cases	0.584	0.265	4.855	0.028^*^	1.792	1.067–3.012
Age	0.017	0.006	7.673	0.006^*^	1.018	1.005–1.030
Duration of symptoms^a^	0.021	0.011	3.764	0.052	1.022	1.000–1.044
Albumin-globulin ratio	−1.720	0.388	19.652	<0.001^*^	0.179	0.084–0.383

CI: confidence interval; CT: computed tomography. ^a^1/100 of the original data; ^*^Significant influence.

Logit (P) = SCORE ≈ 0.706 + 1.413 × [Dust exposure (no = 0, yes = 1)] + 0.584 × [Patient classification (initial treatment = 0, retreatment = 1)] + 0.017 × age − 1.720 × albumin-globulin ratio.

There were significant differences in score variable (χ^2^ = 97.820, P < 0.001), and the fitting degree was good (*χ*^2^ = 4.737, P = 0.785), and the prediction accuracy was 71.5%.

### ROC analysis and diagnostic value of different factors

The ROC analysis results of age, albumin-globulin ratio and combined score were summarized in Table 5, and the cutoff values were 39 years, 0.918 and −0.085, respectively. The AUC value of the combined score was up to 0.753, and the sensitivity was 53.93%, and the specificity was 83.16%, and the positive predictive value was 66.67%, and the negative predictive value was 74.92%. The diagnostic efficiency was further improved than that of a single independent risk factor (Table 5, Fig. 1d and Table S2) .

**Table 5:** Receiver operating characteristic analysis results of risk factors and score.

Variable	AUC (95% CI)	*P* value	Youden index	Cutoff value	Se	Sp	PPV (%)	NPV (%)
Dust exposure	0.633 (0.582–0.683)	<0.001	0.266	NA	0.876	0.390	47.27	83.46
Classification of cases	0.597 (0.545–0.649)	<0.001	0.194	NA	0.814	0.379	45.03	76.60
Age	0.627 (0.573–0.681)	<0.001	0.281	39.00	0.449	0.832	62.50	70.75
Albumin-globulin ratio	0.652 (0.601–0.703)	<0.001	0.258	0.918	0.697	0.561	49.80	74.77
Score	0.753 (0.708–0.798)	<0.001	0.371	−0.085	0.539	0.832	66.67	74.92

AUC: area under the curve; CI: confidence interval; NA: not available; NPV: negative predictive value; PPV: positive predictive value; Se: sensitivity; Sp: specificity.

## Discussion

In the present investigation, the factors that may affect severe lesions were analyzed from the aspects of medical history and laboratory examination based on the classification criterion of mild to moderate lesions and severe lesions by chest CT examination results in APTB patients, in which the independent risk factors affecting severe lesions were successfully screened out and the combined score was constructed, providing a relatively convenient, economical and rapid evaluation method, and also providing a supporting basis for the basic clinical features to assist in the assessment of the severity of pulmonary lesions.

The data in this research demonstrated that the extensive intrapulmonary lesions accounted for 61.56% in the diagnosed APTB, which was similar to that reported by Kombila *et al*. (71.21%)^16^. In the patients with extensive lung lesions, 72.28% (206/285) of whom was mainly cavity type lesion and 56.31% of whom was mostly thick-walled cavity (56.31%). This was similar to the previous study^17,18^ which reported that sputum positive pulmonary tuberculosis had a good correlation with cavity, while the thick wall was composed of fiber tissue, inflammatory granulation tissue and caseous necrotic substance, and the thickness of the wall was positively correlated with the sputum bacterial load^19^. The existence of this lesion type and other risk factors may be one of the pathological bases for the easier progress of intrapulmonary lesions. There was no difference in the amount of sputum bacterial load between the mild to moderate lesions and the severe lesions in this study, and this classification based on the area of the lesion could still not be completely equivalent to the relationship between the nature of the lesion and the amount of sputum.

After exposure to silica dust during quarrying, the powder was mainly distributed in the middle zone of the middle and upper lung fields of both lungs, and CT examination was more sensitive to the detection of this kind of small shadow. The present research revealed that the patients with a history of dust exposure had a greater risk of severe lung lesions, but there was no difference in the number of CD4 and CD8 T lymphocytes in the peripheral blood and the sputum bacterial load (Table S3). There was no significant correlation between the cumulative dust exposure years and the lesion degrees (Fig. 1b), and it also had no correlation with the number of CD4, CD8 T lymphocytes and sputum bacterial load (Fig. S1), suggesting that the history of dust exposure and the cumulative exposure years does not affect the number of peripheral blood T lymphocytes and sputum bacterial load, and it is more likely that the infection of *Mycobacteria Tuberculosis* caused by the history of the dust exposure may be related to the decrease of the ability of scavenging particles by the change of macrophage function caused by silica dust, and the change of local immune mechanism^20,21^, while the characteristics of dust Brownian movement may be more characterized by diffusive lesions of both lungs.

Earlier studies reported that the incidence of severe lung lesions in smokers was higher^8–10^, and the present investigation further supported this theory of “Negative effects of smoking on CT findings” by the change in the cumulative amount of smoking (the smoking index) (Fig. 1a) . When weighted in the model, the effect of smoking rate on severe lesions was not significant, so it eventually was not included in the model. In addition, there was no significant difference in the incidence of diabetes between the two different lesion groups, but the patients with extensive lesions of imaging had higher levels of glycosylated hemoglobin (Fig. 1c), which was similar to the previous report^13^.

The proportion of severe pulmonary lesions in the patients with retreatment was 76.60% (108/141) in this study, which was lower than that of Cohen *et al*.^22^ that there were 88% of the retreatment patients with chronic pulmonary disease by employing a prospective method. It may be more widely related chronic lesions (such as chronic obstructive pulmonary disease, etc.) were included in previous studies. The patients with retreatment were more likely to have severe CT lesions, which may be related to the factors such as longer disease duration, easily spread lesions and drug resistance.

Age increase was an independent risk factor for severe intrapulmonary lesions. The incidence of severe intrapulmonary lesions in the elderly (≥60 years) (20.95%, 97/463) was statistically significant compared with other age groups in this study, which is similar to that of Righi *et al*.^23^. The reason may be that the elderly patients had a long course of disease and recurrent attack of pathological changes, which showed that the new and old lesions existed at the same time, and they were more likely to have disseminated tuberculosis, resulting in a wider distribution of CT lesions. The cutoff value for severe intrapulmonary lesions was 39 years, which could be associated with a significant increase in the rate of exposure to dust at the age of 40–49 in this study (Fig. 1e, Table S4a–c), indicating that not only it should be highly alert to the increased tendency of severe intrapulmonary lesions in elderly patients with pulmonary tuberculosis, but also the superimposed effect of dust exposure may advance the age of severe pulmonary lesions.

As an alternative index of nutritional status of the body, albumin mostly decreased in pulmonary tuberculosis patients^24^. Globulin contains a1 anti-pancreatic protein, a1 acid glycoprotein, complement C3, complement C4, C reactive protein and many acute phase reaction protein components, and most of them show upward changes in pulmonary tuberculosis^25,26^. The decrease rate of albumin (<35 g/L) was 75.38% (349/463) in this study, while the decrease rate of albumin-globulin ratio (<1.5) was 94.99% (435/458). The results of the multivariate analysis showed that the albumin-globulin ratio was the most negative factor, which might indicate that the nutritional status and the inflammatory response of the albumin-globulin ratio had more potential to respond to the pathological changes in the lung. As other studies have shown, albumin in pulmonary tuberculosis decreased significantly with the increase of the percentage of lung field area involved^7^. In addition, it was found in univariable analysis that other significant factors (such as duration of symptoms, platelets, etc.) lost their influence in the model because of their limited contribution, while T lymphocytes count and other factors without statistical significance still needed to be further studied.

The limitation of this study is as follows: (1) The present investigation is an analysis of the hospitalized patients, but only fewer cases (3 cases) in mild lesions were included, so there is the inevitable selectivity bias, which may affect the sensitivity of the combined score. (2) Some medical history may need to be further refined, such as the number of confirmed pneumoconiosis cases in the history of dust exposure, which is mainly limited to the difficulty of pneumoconiosis diagnosis. In this study, there is no further subgroup classification of the pneumoconiosis. (3) The extension of the research results should be employed in clinical diagnosis or suspected pulmonary tuberculosis cases with caution. However, this study further optimized the clinical predictors for the identification of severe lesions in chest CT, and the diagnostic value was improved by one level through the constructed combined score.

## Conclusions

Dust exposure history, retreatment patients, age increase and low albumin-globulin ratio are independent risk factors for chest CT examination of severe lesions in the patients with APTB. Such patients should be alert to the possibility of extensive lesions in lungs. Compared with that of the single factor, the constructed combined score in this study has relatively better diagnostic efficiency. Therefore, the study can provide important help to doctors and nurses in resource-constrained areas.

## Materials and Methods

This study was approved by the Ethics Committee of the Ankang Central Hospital (ECACH-2016013). The clinicl case review would not expose the patients to dangers. Therefore, the Ethical Committee of the hospital further approved that informed consent was not necessary. All the experimental protocol and the methods were performed in accordance with relevant guidelines and regulations, and complied with the principles of the Declaration of Helsinki.

### Study populations

**Figure 2:**
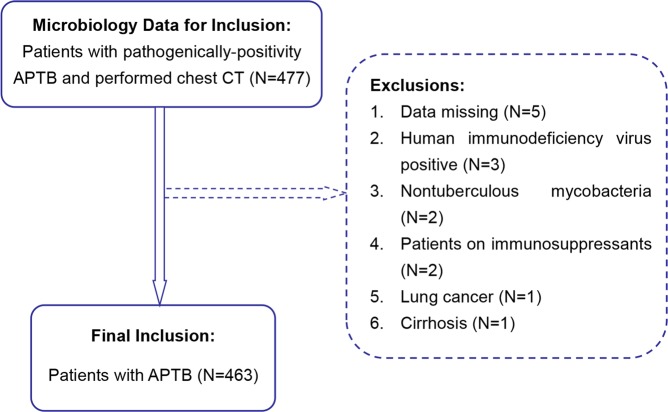
Flowchart of the study population. APTB: Active pulmonary tuberculosis; CT: Computed tomography.

The clinical data of the diagnosed patients with APTB who were hospitalized and examined by chest CT from October 1, 2016 to June 30, 2018 were collected from the Image Storage and Transmission System workstation. The inclusion and exclusion cases were shown in (Fig. 2). The final 463 cases with 363 males and 100 females were included in the analysis. The age ranged from 7 to 86 years, with an average of [50 (36 to 63)] years.

### Imaging protocol

The patients received the GE LightSpeed VCTXT 64-slice spiral CT chest scanning (GE, Boston, Massachusetts, USA), and the technical parameters could be found in the Supplemental Materials (Table S5).

### Imaging analysis

**Figure 3:**
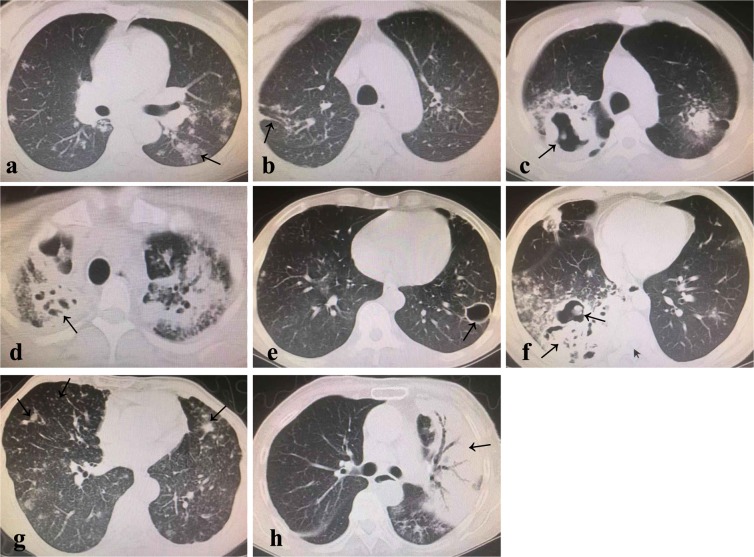
Patch-like shadow (**a**), filamentous shadow (**b**), thick-walled cavity (**c**), mouth-eaten cavity (**d**), thin-walled cavity (**e**), thick wall + mixed mouth-eaten mixed cavity (**f**), speckled nodular shadow (**g**), cheese-like lesion (**h**).

(1) Chest CT was firstly reported by an imaging technician, and then another imaging technician checked, and finally a tuberculosis clinician judged again after fetching the data. If the results were still inconsistent, the expert group would re-read the image, and reached a consensus after discussion. (2) Image classification criterion^27^ of the chest CT was as follows: Stage 1 (minimal/mild): Mild to moderately dense lesions with no cavity and involving only part of one lung or both lungs, The entire range is smaller than the volume of the lung on one side above the junction between the second rib and the sternum. Stage 2 (moderate): Lesions were in one lung or both lungs, but the entire range did not exceed any of the following: ① Small or moderate diffuse lesions with a distribution that did not exceed the entire area of one lung. if lesions were in both lungs, the total area of the lesions did not exceed the area of one lung; ② Highly dense fusion lesions that did not exceed one-third of the volume of a single lung; ③ When there were cavities, the largest diameter of the cavity was less than 4 cm. Stage 3 (advanced): Cases in which the lesion range exceeded the described above for moderate lesions. (3) Lesion types (Fig. 3): Patch-like shadow: the high-density shadow can be seen in the lung, which is patchy and cloudy, with irregular shape, uneven density, and blurred or clear edges. Filamaentous shadow: the lung has a high density shadow that changes in the shape of stripe, similar to the shape of one or more tiny chains. Speckled nodular shadow: uniform/uneven distribution, uniform/uneven size, uniform/uneven density, rounded or oval miliary nodular shadow with a diameter of 1-3 mm; calcification in some lesions, which could be complicated with hilar and mediastinal lymph nodes swelling. Cheese-like lesion n: it is manifested as the lobar consolidation, with multiple small voids inside, as well as disseminated pulmonary tuberculosis along the airway.

(4) Cavity types (Fig. 3): Cavity wall ≥3 mm was thick wall, and cavity wall <3 mm was thin wall. A plurality of bright areas of different sizes and irregular shapes in the lobi pulmonis and the segment sheet-like real image, and no clear wall was mouth-eaten cavity, and more than two types of cavities coexisted as mixed cavity.

### Measurement of other variables

Smoking referred to ≥1 cigarette of daily smoking and ≥3 months, and the smoking index was the number of cigarettes smoked per day multiplied by the number of years of smoking (year × each/d). The history of dust exposure refered to the duration of contact with solid particles ≥3 months in the process of stone mining. The criterion for diagnosis of diabetes was random blood glucose ≥11.1 mmol/L or fasting blood glucose ≥7.0 mmol/L^28^. Terms related to tuberculosis were in accordance with the report of “Diagnostic Criteria and Principles for the Management of Infectious Pulmonary Tuberculosis”^29^, and the classification criteria for sputum bacterial load were provided in the Table S6. Test platform and the associated lymphocyte detection kit (Multitest CD3/CD8/CD45/CD4, BD Biosciences, Franklin Lakes, NJ, USA) were employed for the detection of T-lymphocytes by flow cytometry (Mindset flow cytometer BriCyte E6, Shenzen Mindray Bio-Medical Electronics Co., Ltd. Shenzhenen, China).

### Statistical analysis

SPSS software (version 22.0, IBM Corp., Armonk, NY, USA) was employed in the present investigation. The measurement data with normal distribution were demonstrated as $\bar{{\rm{x}}}$ ± *s*, and the measurement data with abnormal distribution data were demonstrated as *M*(*P*_25_ − *P*_75_). Statistically significant variables were selected by employing the *χ*^2^-test, t-test, and the Mann-Whitney U rank sum test. After the missing values were replaced by a series of mean values, the statistically significant variables by univariate analysis (adjusting test level P < 0.20) were included in the multivariate logistic regression analysis (Forward LR method). The odds ratio (OR) was employed to describe the correlation intensity between the corresponding variables and the severe lesions of CT results, and the 95% confidence interval was listed. The fitting degree of the score was tested by Hosmer-Lemeshow Test (HL test); and the receiver operating characteristic (ROC) curve was employed to determine the cutoff value of the quantitative variables in the independent risk factors (The value corresponding to the sum of the highest sensitivity and specificity). The difference of area under the curve (AUC) was compared by DeLong method using MedCalc software (version 19.0.4, Solvusoft Corp., Las Vegas, NV, USA). P < 0.05 indicated that there was significant difference.

### Study subjects or cohorts overlap

Some study subjects or cohorts have been previously reported in *BMC infectious diseases* (10.1186/s12879-019-4310-y).

## Supplementary information

Supplementary Materials.
