# Supplementary material for: A valuable computed tomography-based new diagnostic tool for severe chest lesions in active pulmonary tuberculosis: combined application of influencing factors
Source: Sci Rep. 2020 Feb 6;10:2023. doi: 10.1038/s41598-020-59041-z (PMC7005193; doi:10.1038/s41598-020-59041-z)
Supplement: Supplementary file 1 — Supplementary Materials. [file 41598_2020_59041_MOESM1_ESM.doc]

**Supplementary information**

**A valuable computed tomography-based new diagnostic tool for severe chest lesions in active pulmonary tuberculosis: combined application of influencing factors**

Kui Li^1,2^, Zicheng Jiang^2^, Yanan Zhu^3^, Chuanqi Fan^2^, Tao Li^2^, Wenqi Ma^4^ & Yingli He^1*^

*^1^Department of Infectious Diseases, The First Affiliated Hospital of Xi'an Jiaotong University, 277 West Yanta Road, Xi'an, 710061, Shaanxi, China.*

*^2^Department of Infectious Diseases, Ankang Central Hospital, 85 South Jinzhou Road, Ankang, 725000, Shaanxi, China.*

*^3^The Medical Imaging Centre, Ankang Central Hospital, 85 South Jinzhou Road, Ankang, 725000, Shaanxi, China.*

*^4^Department of Ultrasound, The Second Affiliated Hospital of Xi'an Jiaotong University, 157 West 5 Road, Xi'an, 710004, Shaanxi, China.*

**Correspondence: heyingli2001@163.com*

| **Table S1.** The comparison results between the smoking index, the life of dust exposure and glycosylated hemoglobin in classification of chest CT lesions. | | | | | | |
| --- | --- | --- | --- | --- | --- | --- |
|  | Stage 1, 2 APTB | | Stage 3 APTB | | Test value | *P* value |
|  | n | M (IQR) or mean ± s | n | M (IQR) or mean ± s |  |  |
| Smoking index, years*each/d | 88 | 400(185－600) | 190 | 600(300－800) | Z = 10412.00 | 0.001 |
| Dust exposure, years | 22 | 9.00(2.75－10.00) | 111 | 10.00(5.00－10.00) | Z = 1391.00 | 0.295 |
| Glycosylated hemoglobin, % | 21 | 8.89 ± 2.18 | 22 | 10.64 ± 2.05 | T =－2.699 | 0.010 |
| APTB: active pulmonary tuberculosis; IQR: interquartile range; M: median. | | | | | | |

| **Table S2.** Comparison of the AUC values of variables individually and in combinations | | | | |
| --- | --- | --- | --- | --- |
| Parameter | AUC difference | Standard error | Z statistic | *P* value |
| Score vs. Dust exposure | 0.120 | 0.035 | 3.457 | 0.0005 |
| Score vs. Classification of cases | 0.156 | 0.036 | 4.398 | ＜0.0001 |
| Score vs. Age | 0.126 | 0.036 | 3.477 | 0.0005 |
| Score vs. Albumin-globulin ratio | 0.101 | 0.025 | 2.910 | 0.0036 |
| AUC: area under the curve. | | | | |

| **Table S3.** Comparison results of T lymphocytes and sputum bacterial loads between dust exposure state. | | | | |
| --- | --- | --- | --- | --- |
|  | Not exposed  (n = 285) | Exposed to dust  (n = 106) | Test value | *P* value |
| CD4 T-cell count, cells/μL, M (IQR) | 304.0 (205.5－397.0) | 319.5 (231.3－439.8) | Z = 16254.50 | 0.247 |
| CD8 T-cell count, cells/μL, M (IQR) | 268.0 (175.5－377.5) | 258.5 (171.3－346.0) | Z = 14400.00 | 0.478 |
| Grade ^a^, N (%) |  |  | χ^2^ =7.028 | 0.219 |
| DNA/RNA positive ^b^ | 75 (26.3) | 21 (19.8) |  |  |
| The number of colony | 19 (6.7) | 3 (2.8) |  |  |
| 1+ | 71 (24.9) | 30 (28.3) |  |  |
| 2+ | 45 (15.8) | 15 (14.2) |  |  |
| 3+ | 52 (18.2) | 22 (20.7) |  |  |
| 4+ | 23 (8.1) | 15 (14.2) |  |  |
| ^a^ Smear grading before treatment; ^b^ Mycobacterium tuberculosis deoxyribonucleic acid or ribonucleic acid positive. | | | | |

| **Table S4a.** Trend of incidence of severe lesions of chest CT and dust exposure rate with age (*N* = 463). | | | | |
| --- | --- | --- | --- | --- |
| Age, years | Chest CT with stage 3 lesions | | Dust exposure | |
|  | n | % (95% CI) | n | % (95% CI) |
| ＜30 | 28 | 6.05 (4.02－8.74) | 1 | 0.22 (0.01－1.20) |
| 30－39 | 25 | 5.40 (3.49－7.97) | 16 | 3.46 (1.98－5.61) |
| 40－49 | 67 | 14.47 (11.21－18.38) | 50 | 10.80 (8.02－14.24) |
| 50－59 | 68 | 14.69 (11.40－18.62) | 40 | 8.64 (6.17－11.76) |
| ≥60 | 97 | 20.95 (16.99－25.56) | 26 | 5.62 (3.67－8.23) |
| CI: confidence interval. | | | | |

| **Table S4b.** Comparison results of the incidence of severe pulmonary CT lesions in different age groups. | | | | | |
| --- | --- | --- | --- | --- | --- |
| Age, years | ＜30 | 30－39 | 40－49 | 50－59 | ≥60 |
|  | *P* value | *P* value | *P* value | *P* value | *P* value |
| ＜30 | － | － | － | － | － |
| 30－39 | 0.680 | － | － | － | － |
| 40－49 | 0.0001 | ＜0.0001 | － | － | － |
| 50－59 | ＜0.0001 | ＜0.0001 | 0.931 | － | － |
| ≥60 | ＜0.0001 | ＜0.0001 | 0.019 | 0.024 | － |

| **Table S4c.** Comparative results of dust exposure rate in different age groups. | | | | | |
| --- | --- | --- | --- | --- | --- |
| Age, years | ＜30 | 30－39 | 40－49 | 50－59 | ≥60 |
|  | *P* value | *P* value | *P* value | *P* value | *P* value |
| ＜30 | － | － | － | － | － |
| 30－39 | 0.0003 | － | － | － | － |
| 40－49 | ＜0.0001 | ＜0.0001 | － | － | － |
| 50－59 | ＜0.0001 | 0.0013 | 0.2918 | － | － |
| ≥60 | ＜0.0001 | 0.1228 | 0.0059 | 0.0848 | － |

| **Table S5.** Technical parameters of chest CT scan | | |
| --- | --- | --- |
| **Items** |  | **Parameters** |
| Scan type |  | Spiral scanning |
| Scanning sequence |  | Chest → routine |
| Exposure condition |  | Tube voltage: 120 kV  Tube current: 250 mA  Rotation time: 0.4 sec  Sweep time: 2.86 sec |
| Field of view |  | Displaying field of view：36.0 cm |
| Matrix |  | 512 × 512 |
| Window width |  | 1500 HU |
| Window level |  | – 500 HU |
| Slice thickness |  | 0.5 mm |
| Slice gap |  | 5.0 mm |
| Reconstruction interval |  | 5.0 mm |
| Pitch |  | 0.984︰1 |
| Rotational speed |  | 0.4 sec |
| Scan rack tilt angle |  | 0^0^ |

| **Table S6.** Smear grading report standard^a^ |
| --- |
| Sputum smear |
| +: 1–9 bacteria/50 fields;  1+: 10–49 bacteria/50 fields;  2+: 1–9 bacteria/field;  3+: 10–90 bacteria/field;  4+: ≥ 100 bacteria/field.  At least 50 fields were observed for the 2+ reports and at least 20 fields were observed for 3+ and above results. |
| Sputum culture |
| +: the actual colony count was reported, as the bacterial colony growth was less than 1/4 of the slope surface area;  1+: bacterial colony growth accounted for 1/4 of the slope surface area;  2+: bacterial colony growth accounted for 1/2 of the slope surface area;  3+: bacterial colony growth accounted for 3/4 of the slope surface area;  4+: bacterial colony growth accounted for entire the slope surface area. |
| ^a^ Refers to *Diagnostic Criteria and Principles of Management of Infectious Pulmonary Tuberculosis*（GB15987-1995）*.* |


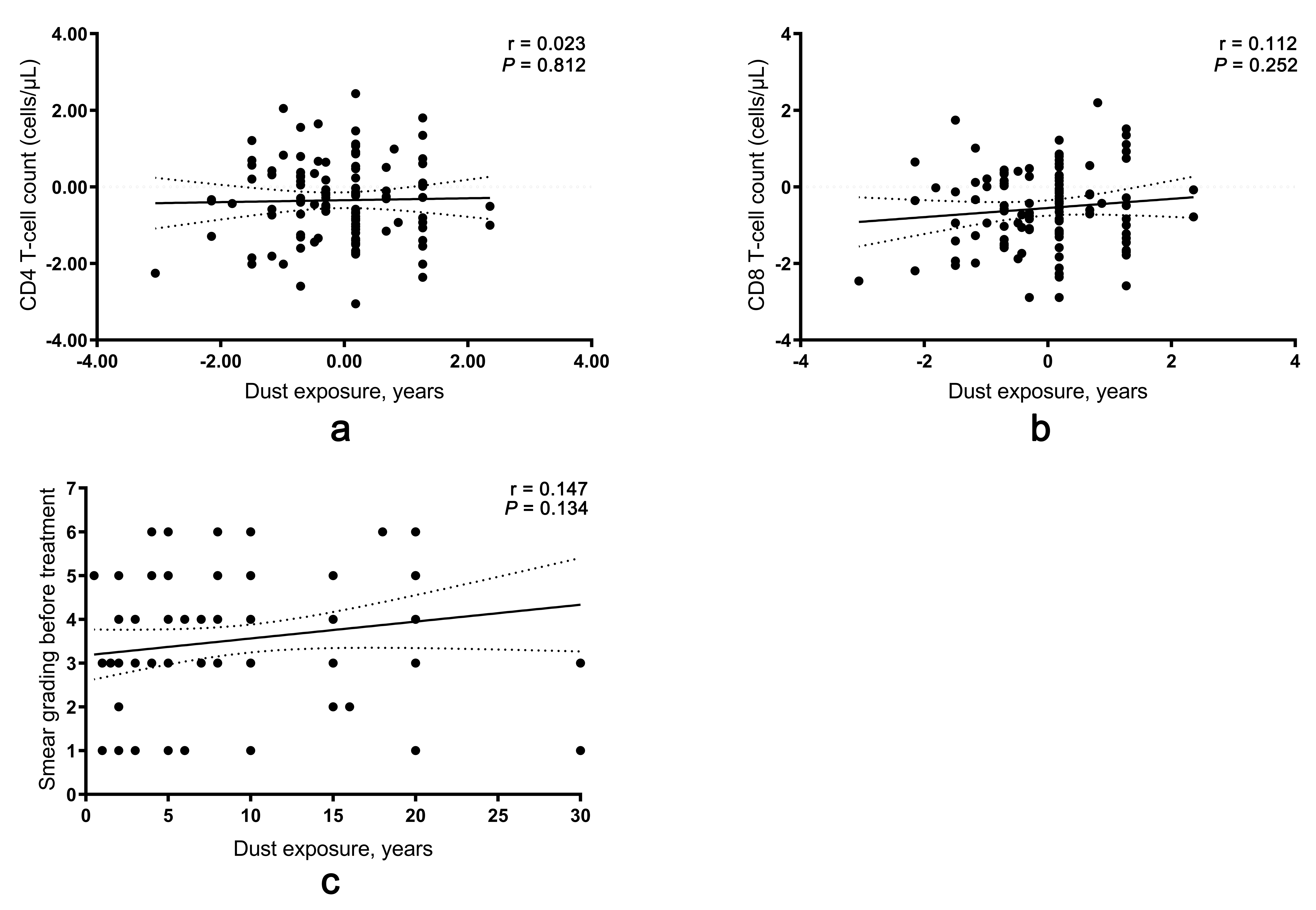


**Figure S1.** The correlation between CD4 T-cell counts and years of dust exposure (a). The correlation between CD8 T-cell counts and years of dust exposure (b), normal integral value by taking Rankit's Formula transformation of CD4 and CD8. The correlation between sputum bacterial load and years of dust exposure (c).
